# Supplementary material for: Acoustic signatures in Mexican cavefish populations inhabiting different caves
Source: PLoS One. 2023 Aug 3;18(8):e0289574. doi: 10.1371/journal.pone.0289574 (PMC10399770; doi:10.1371/journal.pone.0289574)
Supplement: S1 File — (PDF) [file pone.0289574.s001.pdf]

Supplemental Methods file for:

Acoustic signatures in cavefish populations inhabiting different caves

Carole Hyacinthe, Joël Attia, Elisa Schutz, Didier Casane, Sylvie Rétaux

Sound recordings and analyses

For natural field recordings, cavefish were directly recorded in their hosting pools in 6 different natural caves, in the dark. This was done either from 10-12 fish maintained inside a large net (approx. 1m^3 free water volume, Fig.1B) installed in their natural pool, or from freely swimming fish in the case of small natural pools (Tinaja and Chica caves). Hydrophones (Aquarian audio H2a XLR, Anacortes, WA, USA) were connected to portative pre-amplifiers (ART Dual Pre USB, NY 14305, USA) and recorders (Zoom H4n, NY 11788, USA) with SD cards, and recording parameters were adjusted with direct audio listening depending on environmental acoustic characteristics of each cave. They were left on sites for overnight recordings, except in the Molino cave: there, the entrance being a 70 meters vertical pit, the cave could not be visited on two consecutive days and the recording was limited to 1h30, during the afternoon. The four fish taped were nevertheless productive, as 50 Clicks and 50 Serial Clicks could be extracted from this short period for analysis. The natural soundscape in Tinaja (heavy and continuous dripping limestone ceiling) prevented to exploit overnight recordings and made us seek for a quiet pond with specimens on the second day, leading to shorter recording as well (1h30). We therefore analyzed ≤100 sounds from the audio bands from other caves, to equilibrate the comparisons and statistical analyses with the more limited Molino and Tinaja dataset. Of note, for each cave the number of sounds analyzed (50) corresponded to minimum ten times the number of variables studies, i.e., 5 variables for the Serial Clicks. See section below for detailed justification of sample size.

Sounds were extracted by ear from audio bands recordings. Each audio track was carefully scrutinized manually from sonograms magnified at a 3-4 seconds temporal window and further at a 0.2 and 1 second bins (Fig.1D) allowing a visual control of each extracted sound motif while listening from T0 to end, including tracks of 11h. Clicks and Serial Clicks were not extracted in an exhaustive but in a systematical manner: only sounds previously described in the acoustic range of Single Click or Serial Clicks produced by fish in the lab or in the wild were considered, and replicates of each identified sound

motif were randomly extracted. This allowed to increase the stringency and to capture the diversity of Clicks and Serial Clicks for a comprehensive view of each cave soundscape.

Sounds were digitized at 44.1 kHz (16-bit resolution) and analysed using fast Fourrier transform (FFT) with Avisoft SAS Lab Pro 5.2.07 software (Avisoft bioacoustics, Glienicke, Germany) (Bertucci et al., 2010). The acoustic structure of single clicks was analyzed using a set of:

-One temporal parameter, the duration, measured from the envelop of the oscillogram,

-Two parameters measured from the oscillogram: RMS amplitude, and signal to noise ratio (SNR; [RMS amplitude of the signal-RMS amplitude of noise]/RMS amplitude of noise),

-Five spectral parameters obtained from power spectra (FFT, window type: Hann, window size: 512; time overlap: 90%) within a 0-22.5 kHz bandwidth. Spectral parameters were: 1) peak frequency of the frequency spectrum (dominant frequency), 2) first quartile of energy (Q25), i.e. the frequency value corresponding to 25% of the total energy spectrum, 3) second quartile of energy (Q50), 4) third quartile of energy (Q75), and 5) interquartile, i.e. difference between Q75 and Q25.

Serial Clicks were examined using five fine temporal parameters including sound duration, pulse number, mean inter-pulse duration, mean pulse duration, and pulse rate (= sound duration/pulse number) using personal routines developed with R package Seewave (Sueur et al., 2008).

Pulses were considered “Single” if they were of short duration (<20msec) and separated by >1sec interval from the next pulse (threshold defined from the histogram of the inter-pulse durations). After calculating the correlation coefficients between the variables, we excluded highly correlated variables ($r > 0.65$ or < -0.65), retaining three uncorrelated variables for Single Clicks (duration, SNR, dominant frequency) and five uncorrelated variables for Serial Clicks. A principal component analysis (PCA; R package FactoMineR) was performed using the 3 variables retained for Single Clicks and the 5 variables retained for Serial Clicks, allowing to draw the centroïds of the 6 caves, surrounded by their 95% confidence circles (e.,g., Fig.2A). A permutated discriminant function analysis (pDFA; R routine from Bertucci et al. 2010 (Bertucci et al., 2010)) performed on the principal components axis of the previous PCA provided a classification procedure that assigned each sound to its appropriate cave (correct assignment) or to one of the others (incorrect assignment) (e.,g., Fig. 2B, insets).

Acoustic distances between caves plotted on a plan according to their actual GPS coordinates were calculated using an agglomerative hierarchical clustering on principle components (Euclidean metric, Ward method), for Single Clicks (Fig. 2C).

An unsupervised hierarchical clustering method was used on the Single Click Molino dataset and displayed on the ACP plan to estimate a potential individual acoustic signature in this cave (Fig.2D).

Sample size

The sample size (n = 50) was chosen to cope with different constraints and to respond to different statistical issues.

(1) The first one was the precision of parameters' estimation for the acoustic variables.

We first considered the Subterráneo cave. We extracted by ear 90 Single Clicks. We considered this sample as representative of all sounds. Then we built several datasets of different sample size (n varying from 30 to 70), by randomly sampling within the 90 sounds. We calculated the variance of the different datasets for the different variables (SNR, dominant frequency, duration). The variance, measuring the precision of the estimation, depended on the sample size: the larger the size, the smaller (the better) the variance. We chose n = 50 as a good compromise between the precision of the estimation and the time needed to extract and analyse the sounds. We kept the 90 sounds for the Subterráneo cave and we analysed a minimum of 50 Single Clicks for the other caves.

(2) In the Molino and Tinaja caves, it was difficult to obtain long recordings: duration was only 1h30. We were able to extract 50 correct Single Clicks, no more, from each recording. This corresponded to the chosen sample size.

(3) The sample size also fitted the needs of the PCA and pDFA. The recommendation in the literature is to use from 3 to 20 times more observations (n) than variables (p) [see for instance Mundfrom, D.J., Shaw, D.G., & Ke, T.L. (2005). Minimum sample size recommendations for conducting factor analyses. International Journal of Testing, 5 (2), 159-168]. It is the case here: for Single Clicks, we have 3 variables, and we have at least 50 sounds per cave (n/p = 17); for Serial Clicks, we have 5 variables, and we have at least 40 sounds per cave (n/p = 8).

(4) For the lab sounds, the automatic method allowed to extract more than 50 sounds per group (4 groups of lab-raised fish originated from four different caves). We compared the results for the complete data and for a set of 50 sounds randomly chosen within the complete dataset. For instance, we had 614 Single Clicks in total for the 4 groups. We randomly extracted 50 Single Clicks in each group (200 sounds in total). We obtained very close results, notably for the PCA (see below), which comforted us in our sample size choice (n = 50) for the field data:

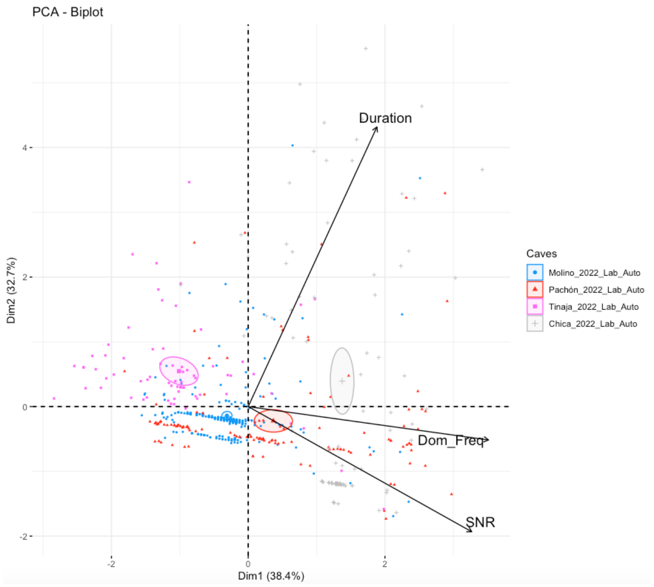

1. PCA on Single Clicks (lab sounds):

614 sounds, original full dataset

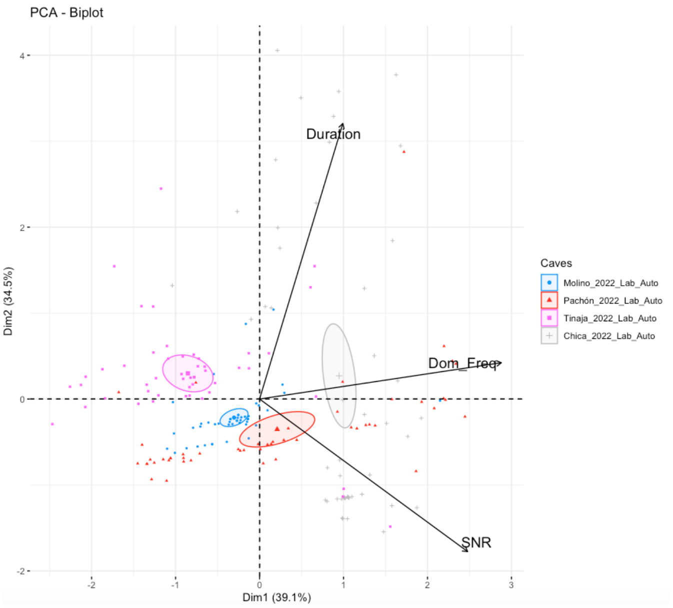

2. PCA on Single Clicks (lab sounds):

200 sounds, re-sampled within the 614 original dataset, 50 per group

Statistics

Normality was assessed with a Kolmogorov-Smirnov test. Kruskal-Wallis tests followed with Dunn's *post hoc* were performed on non-normally distributed data sets. In figures, box plots show the distribution, median and extreme values (top and bottom whiskers) of samples. Statsoft Statistica 6, GraphPad Prism 9 and R 3.1.3 (R Development Core Team, 2016) were used for statistical analyses and graphical representations.
