# Supplementary material for: Acoustic signatures in Mexican cavefish populations inhabiting different caves
Source: PLoS One. 2023 Aug 3;18(8):e0289574. doi: 10.1371/journal.pone.0289574 (PMC10399770; doi:10.1371/journal.pone.0289574)
Supplement: Attachment — Submitted filename: Response to reviewers.pdf [file pone.0289574.s003.pdf]

PONE-D-23-18782
Acoustic signatures in Mexican cavefish populations inhabiting different caves

Journal Requirements:

When submitting your revision, we need you to address these additional requirements.

1. Please ensure that your manuscript meets PLOS ONE's style requirements, including those for file naming. The PLOS ONE style templates can be found at https://journals.plos.org/plosone/s/file?id=wjVg/PLOSOne_formatting_sample_main_body.pdf and https://journals.plos.org/plosone/s/file?id=ba62/PLOSOne_formatting_sample_title_authors_affiliations.pdf

Done. The ms has been formatted according to requirements, and figures and supplementary material have been renamed.

2. We note that the grant information you provided in the 'Funding Information' and 'Financial Disclosure' sections do not match. When you resubmit, please ensure that you provide the correct grant numbers for the awards you received for your study in the 'Funding Information' section.

I do not understand this comment. To me, funding acknowledgements were the same in the online submission system and in the text/acknowledgements. As requested, it has been deleted from the text.

3. Thank you for stating the following in the Acknowledgments Section of your manuscript:
"Work supported by a Lidex Neuro-Saclay collaborative grant to SR and JA, an Equipe FRM grant (DEQ20150331745) to SR, and an Ecos-Nord exchange Program (M15A03) to SR and Patricia Ornelas-Garcia. Fondation des Treilles prize fellowship to CH.
We thank Luis Espinasa, Julien Fumey, Stéphane Père and all members of the Rétaux's lab for their helpful spirit in the field, Patricia Ornelas-Garcia for obtaining shared fieldwork permits, Brian Martineau and the aquatic facility of the Tabin lab for animal care and Joshua Gross for his donation of the Chica cavefish. We thank Clifford Tabin for help and support. "

We note that you have provided funding information that is not currently declared in your Funding Statement. However, funding information should not appear in the Acknowledgments section or other areas of your manuscript. We will only publish funding information present in the Funding Statement section of the online submission form.

As requested, funding acknowledgements have been deleted from the text.

Please remove any funding-related text from the manuscript and let us know how you would like to update your Funding Statement. Currently, your Funding Statement reads as follows:
'Work supported by:
a Lidex Neuro-Saclay collaborative grant to SR and JA (no website),
an Equipe FRM grant (DEQ20150331745) to SR (https://www.frm.org/),
an Ecos-Nord exchange Program (M15A03) to SR and Patricia Ornelas-Garcia

(https://www.univ-spn.fr/ecos-nord/).
a Fondation des Treilles prize fellowship to CH (https://www.les-treilles.com/).

The funders had no role in study design, data collection and analysis, decision to publish, or preparation of the manuscript."

This funding statement as written above is correct and complete.

Please include your amended statements within your cover letter; we will change the online submission form on your behalf.

Done

4. Please amend your list of authors on the manuscript to ensure that each author is linked to an affiliation. Authors' affiliations should reflect the institution where the work was done (if authors moved subsequently, you can also list the new affiliation stating "current affiliation:…." as necessary).

Done

5. We note that Figure 1a in your submission contain [map/satellite] images which may be copyrighted.

No, the schematic map in Fig 1A is an original drawing that I did myself, and is in no way copyrighted by Google maps or any other website.

6. Please include a copy of Table 1 which you refer to in your text on page 6.

Done. Sorry for the mistake in the initial submission. It has been inserted in text, according to PlosOne formatting instructions.

7. Please include captions for your Supporting Information files at the end of your manuscript, and update any in-text citations to match accordingly. Please see our Supporting Information guidelines for more information: http://journals.plos.org/plosone/s/supporting-information.

Supporting information does not need captions. It contains only Supp Methods (S1_file) and a raw data excel file (S2_table). There are no figures.

8. Please review your reference list to ensure that it is complete and correct. If you have cited papers that have been retracted, please include the rationale for doing so in the manuscript text, or remove these references and replace them with relevant current references. Any changes to the reference list should be mentioned in the rebuttal letter that accompanies your revised manuscript. If you need to cite a retracted article, indicate the article's retracted status in the References list and also include a citation and full reference for the retraction notice.

Done

Reviewers' comments: Reviewer's Responses to Questions

Comments to the Author

1. Is the manuscript technically sound, and do the data support the conclusions?

The manuscript must describe a technically sound piece of scientific research with data that supports the conclusions. Experiments must have been conducted rigorously, with appropriate controls, replication, and sample sizes. The conclusions must be drawn appropriately based on the data presented.

Reviewer #1: Yes

Reviewer #2: Yes

---

2. Has the statistical analysis been performed appropriately and rigorously?

Reviewer #1: Yes

Reviewer #2: Yes

---

3. Have the authors made all data underlying the findings in their manuscript fully available?

The PLOS Data policy requires authors to make all data underlying the findings described in their manuscript fully available without restriction, with rare exception (please refer to the Data Availability Statement in the manuscript PDF file). The data should be provided as part of the manuscript or its supporting information, or deposited to a public repository. For example, in addition to summary statistics, the data points behind means, medians and variance measures should be available. If there are restrictions on publicly sharing data—e.g. participant privacy or use of data from a third party—those must be specified.

Reviewer #1: Yes

Reviewer #2: Yes

---

4. Is the manuscript presented in an intelligible fashion and written in standard English?

PLOS ONE does not copyedit accepted manuscripts, so the language in submitted articles must be clear, correct, and unambiguous. Any typographical or grammatical errors should be corrected at revision, so please note any specific errors here.

Reviewer #1: Yes

Reviewer #2: Yes

5. Review Comments to the Author

Please use the space provided to explain your answers to the questions above. You may also include additional comments for the author, including concerns about dual publication, research ethics, or publication ethics. (Please upload your review as an attachment if it exceeds 20,000 characters)

Reviewer #1: The manuscript: “Acoustic signatures in Mexican cavefish populations inhabiting different caves” by Hyacinthe et al. presents a study on the acoustic communication of cavefish populations in different natural caves. The researchers conducted acoustic recordings in six caves and analyzed the sound production of the cavefish populations. They aimed to determine if there were distinct acoustic signatures or "accents" associated with different cave populations and if these signatures were genetically maintained in the laboratory.

The study recorded various types of sounds produced by the cavefish, with a focus on Clicks and Serial Clicks, which showed the largest frequency bandwidth. They extracted acoustic parameters from these sounds and compared them between caves to identify any significant variations.

The results of the analysis revealed that both Clicks and Serial Clicks exhibited significant differences among the cavefish populations in terms of sound duration, dominant frequency, and signal-to-noise ratio. Excitingly, these differences were observed both in the recordings from the wild populations and the laboratory-bred populations, despite differences in their living environments and life experiences, indicating that it is a robust genetic trait.

The findings of this study have important implications for understanding the evolution of acoustic communication and potential speciation in cavefish populations. The presence of distinct acoustic signatures among different cave populations indicates the possibility of acoustic divergence and the development of local accents over time. These acoustic differences could contribute to the reproductive isolation and speciation of cavefish populations which demonstrates the potential for using acoustic analysis as a tool for studying the evolution and speciation of species.

Overall, the paper is well-written and provides detailed information about the methods used, the results obtained, and their implications. The findings contribute to the understanding of acoustic communication in cavefish populations and open up avenues for further research in this field.

Thank you for your nice appreciation of our work.

I have only minor comments:

The abstract does not do justice to the exciting findings of the paper. I recommend revising it to emphasize the identification of distinct cave-specific genetic traits and their preservation within the laboratory setting, indicative of a robust genetic basis.

Done, abstract has been slightly changed accordingly.

Given that the data does not offer conclusive evidence supporting or refuting genetic drift or selection, I propose excluding the discussion on this topic from both the abstract and the main text.

We have removed the words about drift from the abstract. However, we would like to keep it in the Discussion section: we think that given the absence of “logics” in the evolution of cavefish accents, according to either geography or local biotic and abiotic conditions, it is the most parsimonious hypothesis, especially since cavefish populations are small (we have added a recent reference from our team’s work to support this later affirmation). We think it is important to emphasize that not everything evolves through a selective regime, and that the evolution of cavefish accents may be a good example of neutral evolution.

It would have been nice to have surface fish included in the study, however, I understand if this is beyond the scope of this study.

Yes, that is true. However, as the reviewer said here we wanted to concentrate on cavefish diversity.

Reviewer #2: This interesting manuscript examines the acoustical signatures of natural cavefish from the El Abra region of Mexico. Intriguingly, these fish were discovered to produce sounds that may be important for communication among members of the same cave locality. Herein, the authors provide a diverse set of analyses of these acoustic signatures and find that non-captive (i.e., "native") cave populations have specific acoustic signatures, and these signatures are likely subject to drift as their principal evolutionary mechanism explaining differences across cave populations. Overall, this is a very interesting and well-conducted studies that is appropriate for publication. The manuscript is well written and presented, and the structure is nicely organized. Below, I provide my comments for the authors to consider - the fact that many are editorial/discretionary speaks to the quality of the submitted manuscript.

Thank you for your nice appreciation of our work.

Comments:

1. The authors summarize broadly the source of acoustic sounds in other teleost species - is the source of sound generation in cave (or surface) morphs known? (e.g., swim bladder v. stridulation of cranial bones?)

Unfortunately, this is not known. It is now indicated in text.

2. The authors reference 'practical limits in each cave' and 'recording conditions'. Can you clarify precisely how these may impacted the collected data for the reader?

OK. In Methods, we have now added: “For example, the Molino cave being at the bottom of a 70 meter pit, we could not visit the cave 2 days in a row and obtain overnight acoustic recordings; or the water level being too low in the Tinaja perched pool, we could not set up a recording net and therefore fish were recorded by simply hanging an hydrophone over their natural pool.”

3. Please cite the described hybrid origin of Chica fish, as referenced in L190 and L254.

Done

Line 190, reference for Subterraneo hybrids: Bibliowicz et al. 2013.

Line 254, reference for Chica hybrids: Breder 1942; Mitchell 1977.

4. This interesting report focuses largely on the production of sound, but less on the reception of sounds by conspecific members of the same locality. Can the authors speak to a response phenotype, that could potentially be examined, that would connect the production of within-cave sounds to reception by other members within the cave? This would seem to ratify both the importance of sounds for a phenotypic outcome (e.g., breeding?), and reinforce the assertion that sound production/response is specific to individual caves.

OK. We did not initially comment on this, because hypotheses are mostly speculative. To respond to the reviewer's demand, we have now added, in the Discussion:

"The function(s) of acoustic communication among cavefish are mostly unknown. Previously, we have shown that Sharp Clicks, one of the six sounds *Astyanax* can produce, are emitted during chemosensory-driven foraging [13]. The meaning or significance of other sounds including Clicks is unknown, and it will be extremely interesting to test their potential function in foraging, or breeding or any other social interaction or behavior".

Minor, discretionary suggestions:

1. L79 - "northeastern" should not be capitalized. Done
2. L80 - are all the cave-populations blind? My understanding was that Caballo Moro has some purportedly sighted fish. Indeed, Caballo Moro has eyed fish, but they remain in the "open" part of the cave. In the dark part of the cave, fish are troglomorphic. Same thing in Chica: the deepest part of the cave that is in contact with the rio Tampaon has eyed, surface-like fish. We do not think such degree of details should be explained here, as we think it may rather confuse the reader.
3. L130 - "...consisted of a click..." Done
4. L138: "clicks" should be lowercase. Done
5. The sentence ending on L139 ends abruptly. Completed
6. L193: 'pule' should be 'pulse' Done
